# The Structure of the Porcine Deltacoronavirus Main Protease Reveals a Conserved Target for the Design of Antivirals

**DOI:** 10.3390/v14030486

**Published:** 2022-02-27

**Authors:** Fenghua Wang, Cheng Chen, Zefang Wang, Xu Han, Peidian Shi, Kaixuan Zhou, Xiaomei Liu, Yunjie Xiao, Yan Cai, Jinhai Huang, Lei Zhang, Haitao Yang

**Affiliations:** 1 School of Life Sciences, Tianjin University, Tianjin 300072, China; wangfh@tju.edu.cn (F.W.); chengchen@tju.edu.cn (C.C.); zefangwang@tju.edu.cn (Z.W.); hxxhf1y@163.com (X.H.); shipeidian@tju.edu.cn (P.S.); yunjiexiao2016@tju.edu.cn (Y.X.); jinhaih@tju.edu.cn (J.H.); 2 Tianjin International Joint Academy of Biotechnology and Medicine, Tianjin 300457, China; 2120161132@mail.nankai.edu.cn (K.Z.); yuer13971194073@163.com (X.L.); caiyan_86@163.com (Y.C.); 3 Key Laboratory of Industrial Fermentation Microbiology of the Ministry of Education, Tianjin Key Laboratory of Industrial Microbiology, College of Biotechnology, Tianjin University of Science and Technology, Tianjin 300457, China; 4 Shanghai Institute for Advanced Immunochemical Studies and School of Life Science and Technology, Shanghai Tech University, Shanghai 201210, China

**Keywords:** coronaviruses, porcine deltacoronavirus, main protease, broad-spectrum antivirals

## Abstract

The existing zoonotic coronaviruses (CoVs) and viral genetic variants are important microbiological pathogens that cause severe disease in humans and animals. Currently, no effective broad-spectrum antiviral drugs against existing and emerging CoVs are available. The CoV main protease (M^pro^) plays an essential role in viral replication, making it an ideal target for drug development. However, the structure of the *Deltacoronavirus* M^pro^ is still unavailable. Porcine deltacoronavirus (PDCoV) is a novel CoV that belongs to the genus *Deltacoronavirus* and causes atrophic enteritis, severe diarrhea, vomiting and dehydration in pigs. Here, we determined the structure of PDCoV M^pro^ complexed with a Michael acceptor inhibitor. Structural comparison showed that the backbone of PDCoV M^pro^ is similar to those of alpha-, beta- and gamma-CoV M^pro^s. The substrate-binding pocket of M^pro^ is well conserved in the subfamily *Coronavirinae*. In addition, we also observed that M^pro^s from the same genus adopted a similar conformation. Furthermore, the structure of PDCoV M^pro^ in complex with a Michael acceptor inhibitor revealed the mechanism of its inhibition of PDCoV M^pro^. Our results provide a basis for the development of broad-spectrum antivirals against PDCoV and other CoVs.

## 1. Introduction

Coronaviruses (CoVs) are round or oval enveloped viruses with a positive-sense RNA genome [1]. CoVs are among the most dangerous microbiological pathogens that infect mammals, such as humans, mice, cats, and pigs, as well as birds, such as sparrows, and they are responsible for a large number of gastric, enteric and respiratory syndromes [2,3,4,5,6]. In 2003, an outbreak of severe acute respiratory syndrome (SARS) led to an international epidemic, and severe acute respiratory syndrome coronavirus (SARS-CoV) was demonstrated to be the etiological agent [7,8,9,10]. In 2012, a novel CoV, Middle East respiratory syndrome coronavirus (MERS-CoV), was reported in Saudi Arabia [11]. MERS-CoV infection can cause patients to develop acute renal failure. In late December 2019, a novel coronavirus (SARS-CoV-2) was identified in Wuhan, Hubei Province. This infectious pneumonia has spread worldwide, and as of January 2022, more than 318 million people have been infected, and 5.5 million have died from the disease [12]. The ceaseless emergence of new pathogenic CoVs indicates that CoVs remain an enormous threat to public health security. However, at present, no effective broad-spectrum antiviral drugs against existing and emerging CoVs are available.

As demonstrated, the existing zoonotic viruses and viral genetic variants are widely known to be a potential threat to public health. It has been reported that SARS-CoV-2 as well as SARS-CoV and MERS-CoV are likely to have originated from animal reservoirs and crossed species barriers to infect humans [13,14]. In addition, a high percentage of sequence variations among CoV strains and recombination during coinfections have been indicated. Therefore, it is necessary to develop broad-spectrum antivirals against existing and emerging CoVs. CoV main proteases (M^pro^s), also called 3C-like proteases, which play a vital role in the proteolytic pathway in viral replication, are known to be among the most attractive and ideal targets for antiviral drug design [15]. The RNA genome of CoVs encodes two replicase polyproteins (pp1a and pp1ab) and other structural proteins. The polyproteins are cotranslationally cleaved into 16 nonstructural proteins (nsp1 to nsp16) and assemble into membrane-anchored replication machinery for transcription or replication [16]. M^pro^s are responsible for cleavage at no fewer than 11 sites, indicating their main role in virus replication and pathogenesis.

M^pro^s are defined as serine proteases, which have the closest relatives with 3C-like proteinases of potyviruses [17]. M^pro^s always function as dimers and employ conserved cysteine and histidine residues at their catalytic site as charge-relay systems [18,19]. At present, CoVs are generally divided into four genera according to their genome sequences, as follows: *Alphacoronavirus* (including human coronavirus NL63 [HCoV-NL63], human coronavirus 229E [HCoV-229E], porcine epidemic diarrhea virus [PEDV], transmissible gastroenteritis virus [TGEV] and feline infectious peritonitis virus [FIPV]), *Betacoronavirus* (including SARS-CoV-2, SARS-CoV, human coronavirus HKU1 [HCoV-HKU1] and MERS-CoV), *Gammacoronavirus* (infectious bronchitis virus [IBV]), and the newly discovered *Deltacoronavirus* genus. Several three-dimensional crystal structures of M^pro^s have been determined for *Alphacoronavirus*, *Betacoronavirus* and *Gammacoronavirus* [18,19,20,21,22,23,24,25,26,27,28,29]. However, little is known about the M^pro^ of *Deltacoronavirus*, posing a blind spot for broad-spectrum drug design against CoV M^pro^s. Currently, human CoVs have been identified in the genera *Alphacoronavirus* and *Betacoronavirus*. There are not yet reports of CoVs from the genera *Gammacoronavirus* and *Deltacoronavirus* infecting humans. However, it has been demonstrated that porcine deltacoronavirus (PDCoV) can infect cells of human origin by interacting with host aminopeptidase N (APN) via its spike (S) protein, and studies have identified children in Haiti who were positive for PDCoV, which poses a considerable concern to human health with adaptive changes [30,31]. Therefore, it is necessary to investigate the properties of the newly identified *Deltacoronavirus* M^pro^ and its relationship with other CoV M^pro^s to direct rational drug design.

PDCoV is a member of the subfamily *Coronaviridae*, genus *Deltacoronavirus*, which was first discovered in 2012 in Hong Kong and named porcine coronavirus HKU15 [6]. Later, the virus spread nationwide and was gradually detected in the US, Korea and various provinces of China [32,33,34,35]. At present, the reasons for the sudden emergence and origin of PDCoV in pigs remain unclear. Similar to PEDV infections, PDCoV infections cause atrophic enteritis, severe diarrhea, vomiting and dehydration in nursing piglets [36], and infected piglets have a high mortality rate [37]. PEDV and PDCoV can infect pigs alone or jointly; these features result in CoV emergence and reemergence in pigs, causing many pig deaths and a dramatic negative economic impact on the animal husbandry industry, leading to major economic losses [32,38]. In this study, we determined the crystal structure of PDCoV M^pro^ from *Deltacoronavirus* complexed with a peptidomimetic inhibitor with a Michael acceptor, which is the substituent group on the activated α, β unsaturated ester and can undergo Michael addition reaction with nucleophile from the enzyme. The complex structure provided insight into the detailed structural features of PDCoV M^pro^ and revealed the mechanism underlying its inactivation by the Michael acceptor. In addition, we identified a drug target conserved among all alpha-, beta-, gamma- and delta-CoVs. We then obtained two modified inhibitors with improved inhibition activity, and propose that peptidomimetic inhibitors carrying a Michael acceptor warhead are effective against the M^pro^s of all CoVs.

## 2. Materials and Methods

### 2.1. Gene Expression and Protein Purification

The PDCoV M^pro^ coding sequence was cloned into the *Bam*HI and *Xho*I restriction sites of the pET-28b_SUMO vector and then transformed into *Escherichia coli* strain BL21 (DE3). The fusion protein SUMO-PDCoV M^pro^ was purified by Ni-affinity chromatography (GE Healthcare, Uppsala, Sweden) and then cleaved with ULP protease. M^pro^ was further purified using anion exchange chromatography (HiTrap Q, GE Healthcare, Uppsala, Sweden) with a linear gradient from 2.5 to 500 mM NaCl (20 mM Tris-HCl pH 8.0) and size exclusion chromatography (Superdex 75 10/300 GL, GE Healthcare, Uppsala, Sweden) in 10 mM HEPES pH 7.5 and 150 mM NaCl.

### 2.2. Crystallization, Data Collection, Structure Determination and Refinement

Crystals of the complex were obtained by cocrystallization following the incubation of 1 mg mL^−1^ PDCoV M^pro^ and 10 mM N3 in the buffer of 10 mM HEPES pH 7.5 and 150 mM NaCl at 4 °C at a molar ratio of 1:5 for 12 h. The complex was concentrated to 9 mg mL^−1^ and then crystallized by the microbatch-under-oil method at 291 K. The successful crystal growth conditions were 0.1 M sodium citrate (pH 5.1) and 4% (*w*/*v*) polyethylene glycol 6000. Crystals were cryoprotected with 20% glycerol, 0.1 M sodium citrate (pH 5.1) and 4% (*w*/*v*) polyethylene glycol 6000 and flash-frozen in liquid nitrogen. Data were collected at the Shanghai Synchrotron Radiation Facility (SSRF) beamline BL19U1 at 100 K using an ADSC Q315r detector with a wavelength of 0.97923 Å. The crystal belonged to space group *P*6_1_ with unit cell dimensions *a* = *b* = 122.3 Å and *c* = 289.8 Å. Diffraction data were processed with HKL3000 (version 721.3, HKL Research, Inc., Charlottesville, VA, USA) (44). The complex structure was solved by molecular replacement using the structure of PEDV M^pro^ (PDB ID 5GWZ) [27] as a search model through the PHASER [39] program from the CCP4 package [40]. Model building and refinement were performed using PHENIX (version 1.14) [41] and COOT (version 0.8.9) [42]. The *R*_work_ and *R*_free_ of the final model were 19.21% and 24.14%, respectively.

### 2.3. Enzyme Activity and Inhibition Assays

Enzymatic assays were carried out as previously reported [15,28,29]. A fluorogenic substrate of PDCoV M^pro^, MCA-AVLQ↓SGFR-Lys(Dnp)-Lys-NH_2_ (>95% purity, GL Biochem Shanghai Ltd., Shanghai, China), was used to assess enzyme activity by measuring fluorescence intensity with excitation and emission wavelengths of 320 nm and 405 nm, respectively. The assay was performed at 30 °C, and the buffer used consisted of 50 mM Tris-HCl (pH 7.3) and 1 mM EDTA. The *K*_m_ and *k*_cat_ of PDCoV M^pro^ and *K*_i_ and *k*_3_ of N3 were determined according to the methods used in our previous work [15,29]. The values of *K*_i_ and *k*_3_ were obtained following the addition of PDCoV M^pro^. The enzyme and substrate concentration were set at 2 µM and 50 µM, respectively. The inhibitor concentration varied among seven different concentrations (6–24 µM). Data were analyzed with the program GraphPad Prism (version 5.0, GraphPad, San Diego, CA, USA). The enzymatic assay used to test M14 and M25 was similar to that used to test N3.

## 3. Results

### 3.1. Overall Structure

**Figure 1:**
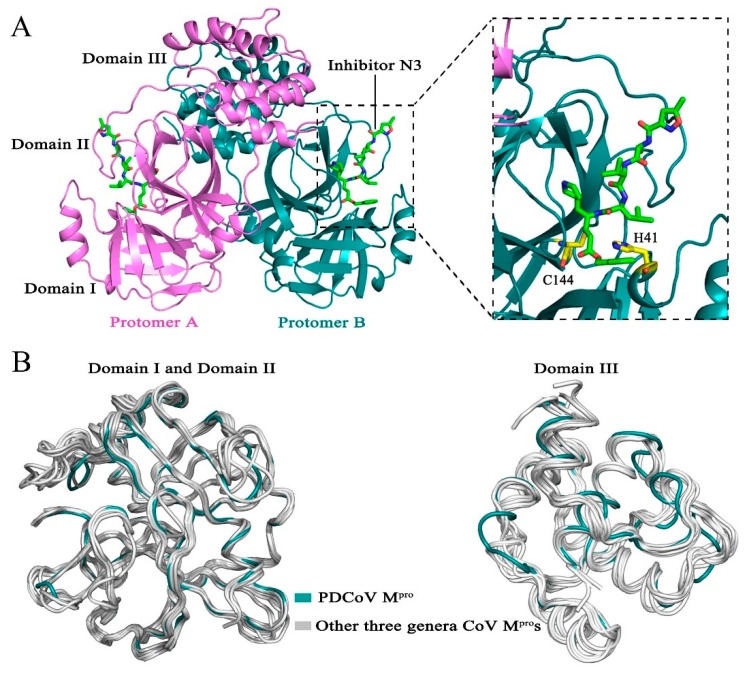
Structure of Porcine deltacoronavirus (PDCoV) main protease (M^pro^**)**. (**A**) Overall structure of the PDCoV M^pro^-N3 complex. The two protomers, A and B, are colored violet and deep teal, respectively; the substrate-binding pocket is indicated by dashed lines; inhibitor N3 is shown as green sticks; and the catalytic dyads Cys-144 and His-41 are colored yellow. (**B**) Superposition of a representative CoV M^pro^ from each of four genera: *Deltacoronavirus* (PDCoV, deep teal) and three other genera (human coronavirus NL63 [HCoV-NL63], human coronavirus 229E [HCoV-229E], porcine epidemic diarrhea virus [PEDV], feline infectious peritonitis virus [FIPV], transmissible gastroenteritis virus [TGEV], severe acute respiratory syndrome coronavirus 2 [SARS-CoV-2], severe acute respiratory syndrome coronavirus [SARS-CoV], Middle East respiratory syndrome coronavirus [MERS-CoV], human coronavirus HKU1 [HCoV-HKU1], mouse hepatitis virus A59 [MHV-A59] and infectious bronchitis virus [IBV], silver).

We cocrystallized PDCoV M^pro^ with a Michael acceptor inhibitor, named N3, and determined the structure of the complex at 2.60 Å resolution (Table 1). The crystal structure contained six M^pro^ molecules per asymmetric unit. In the crystals, two neighboring molecules, protomer A and protomer B, formed a typical homodimer. Each protomer contains three domains: domain I (residues 1–97), domain II (residues 98–186) and domain III (residues 200–304). Domain I and II each have a chymotrypsin-like fold, and domain III is composed of five α-helixes and contributes to the formation of a homodimer (Figure 1A). The substrate-binding pocket, which contains a catalytic dyad (His-41 and Cys-144), is located in the cleft between domains I and II (Figure 1A). The superimposition of M^pro^s [15,21,22,23,24,25,26,27,28,29] from four different CoV genera shows that the PDCoV M^pro^ shares a similar overall structure and backbone with other CoV M^pro^s (Figure 1B). Domain I (residues 1–97) and domain II (residues 98–186) of M^pro^ from PDCoV are well conserved, with Cα root-mean-square deviations (RMSDs) of 1.4–1.7 Å, 1.3–1.4 Å and 1.1 Å in comparison with those of alpha-, beta-, and gamma-CoV, respectively. Structural overlay of the M^pro^s from four CoV genera shows that domain III (residues 200–304) of PDCoV has a similar orientation to that of the other CoV M^pro^s. The Cα RMSDs between different CoVs and PDCoV are summarized in Table 2.

### 3.2. The Substrate-Binding Pocket PDCoV M^pro^ Is Structurally Conserved Relative to M^pro^s from the Other Three Genera

To provide more insight into the properties of PDCoV M^pro^, we analyzed the substrate-binding pocket between domain I and domain II.

**Figure 2:**
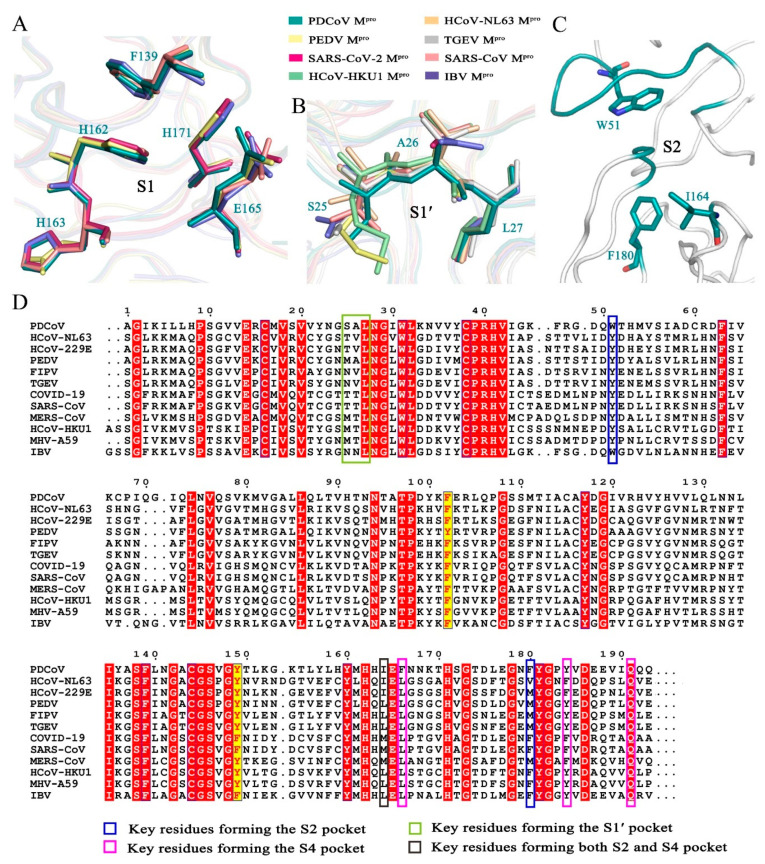
Analysis of the substrate-binding pocket of PDCoV M^pro^. (**A**) Superimposition of the key residues in the S1 binding pockets of PDCoV M^pro^ (deep teal), PEDV M^pro^ (pale yellow), SARS-CoV M^pro^ (salmon) and IBV M^pro^ (slate). (**B**) Superimposition of the key residues in the S1′ binding pocket of PDCoV M^pro^ (deep teal), HCoV-HKU1 M^pro^ (pale green), HCoV-NL63 M^pro^ (light orange), PEDV M^pro^ (pale yellow), SARS-CoV-2 M^pro^ (warm pink), SARS-CoV M^pro^ (salmon), TGEV M^pro^ (silver) and IBV M^pro^ (slate). (**C**) Hydrophobic residues in the S2 binding pocket. (**D**) Sequence alignment of the M^pro^ domains I and II from four different genera. PDCoV from *Deltacoronavirus*; HCoV-NL63, PEDV, FIPV, TGEV from *Alphacoronavirus*; SARS-CoV-2, SARS-CoV, MERS-CoV, HCoV-HKU1, MHV-A59 from *Betacoronavirus*; IBV from *Gammacoronavirus*. A sequence alignment was generated using the program ClustalW and drawn by ESPript3. White letters with red backgrounds show identical residues, and red letters with yellow backgrounds show conservative variation.

The S1 binding pocket of PDCoV M^pro^ is composed of residues Phe-139, His-162, Glu-165, and His-171 and the backbones of the other amino acids, such as Leu-140, Asn-141, His-163 and Ile-164. Sequence analysis showed that the M^pro^ cleavage sites at the P1 position in the identified PDCoV were all glutamines, indicating that the S1 substrate-binding pocket of PDCoV M^pro^ has an extremely strong preference for glutamine residues. In our previously reported structure of the complex between a SARS-CoV M^pro^ H41A mutant and an 11-peptidyl substrate, the Nε2 atom of His-163 and the main chain carbonyl oxygen of Phe-140 in the S1 binding pocket form three hydrogen bonds with glutamine at the P1 position [28]. Structural superposition of the S1 binding pocket in M^pro^s from the CoVs of four genera showed that in PDCoV M^pro^, the carbonyl oxygen atom of Phe-139, the imidazole ring NH of His-162 and the carbonyl oxygen atoms of residues at position 163 in PDCoV M^pro^, PEDV M^pro^, SARS-CoV-2 M^pro^, SARS-CoV M^pro^ and IBV M^pro^ are extremely conserved. In addition, we found that the key residues that form the S1 binding pocket, His-171 and Glu-165, also share a similar structure (Figure 2A). For the above reasons, we concluded that the *Deltacoronavirus* PDCoV M^pro^ shares a conserved S1 binding pocket with M^pro^s from the other three genera. The evolutionary conservation of amino acids plays a crucial role in drug design.

In the structure of PDCoV M^pro^, the side chains of Trp-51, Ile-164 and Phe-180 as well as residues 186–188 and a loop from 41–51 form a deep, hydrophobic S2 binding pocket. Residues 186–188 and a loop from 41–51 contribute to the outer wall of the S2 site (Figure 2C). Furthermore, the side chains of the hydrophobic amino acids Trp-51, Ile-164 and Phe-180 of the S2 subsite interact with residues at the P2 position through hydrophobic interactions. The S2 subsite of CoV M^pro^s usually prefers a hydrophobic residue. Leucine is the most common residue at the P2 site. The M^pro^s of HCoV-NL63 and HCoV-229E from *Alphacoronavirus* recognize Leu/Ile/Val; those of SARS-CoV-2, SARS-CoV, HCoV-HKU1, and MERS-CoV from *Betacoronavirus* seem to prefer Phe/Met/Pro/Leu/Val. The residues at the P2 position of M^pro^ from *Gammacoronavirus* viruses such as IBV are Leu/Met/Val. In *Deltacoronavirus* viruses such as PDCoV, this position favors Val/Leu. The corresponding residues at positions 51, 164 and 180 of M^pro^s from the other three genera of CoVs are extremely similar and are almost always hydrophobic amino acids, such as Phe, Leu, Met, Val, Ile, Trp and Tyr, contributing to the interactions between the binding pocket and substrate (Figure 2D).

The S4 binding pocket of PDCoV M^pro^ is composed of residues 164–167, 183–184 and 188–191. The side chains of Ile-164, Phe-166, Tyr-184, and Gln-191 form the hydrophobic S4 subsite of PDCoV M^pro^. Sequence alignment showed that the conserved hydrophobic amino acids in the S4 binding pocket play a key role in hydrophobic interactions (Figure 2D).

Residues Ser-25, Ala-26, Leu-27, His-41, Val-42, Lys-45 and Asn-141 form the S1′ binding pocket. The side chains of the amino acids at positions 25, 26 and 27 directly interact with the P1′ residue of the substrate via van der Waals interactions [28]. The backbone atoms of the residues that form the S1′ pocket of PDCoV M^pro^ are similar to the corresponding sequences in the other three genera. Sequence alignment showed that the residues at position 25 of M^pro^ from PDCoV, PEDV, SARS-CoV-2, SARS-CoV and IBV are different from the consensus of the CoV M^pro^s from the four genera; these residues are Thr, Met, Asn and Ser, respectively (Figure 2B,D).

### 3.3. The Peptidomimetic Inhibitor N3 Efficiently Inhibits PDCoV M^pro^

We determined the *K*_m_ and *k*_cat_ of PDCoV M^pro^ to be 56.6 ± 1.9 µM and 0.030 ± 0.009 s^−1^, respectively (Table 3). This *K*_m_ value is close to that of HCoV-NL63 M^pro^ (50.8 ± 3.4 µM) and TGEV M^pro^ (61 ± 5 µM), lower than that of mouse hepatitis virus A59 (MHV-A59) M^pro^ (77 ± 5 µM), HCoV-HKU1 M^pro^ (83.2 ± 13.3 µM), SARS-CoV M^pro^ (129 ± 7 µM) and IBV M^pro^ (139 ± 15 µM), and higher than that of HCoV-229E M^pro^ (29.8 ± 0.9 µM) and FIPV M^pro^ (13.5 ± 1.8 µM) [15,26,29] (Table 3). Structural analysis showed that the substrate-binding pocket of PDCoV M^pro^ shares several features in common with M^pro^s from CoVs in the other three genera, especially the S1, S2 and S4 subsites. The key residues at these sites are almost completely conserved (Figure 2). The N3 is a peptidomimetic inhibitor designed against various M^pro^s, such as those from SARS-CoV, HCoV-229E and FIPV [15]. Therefore, we deduced that the Michael acceptor and peptidomimetic inhibitor N3 may inhibit PDCoV M^pro^. An enzymatic assay showed that N3 inactivated PDCoV M^pro^. The calculated *K*_i_ and *k*_3_ were 11.98 ± 0.13 µM and 72.91 ± 7.05 (10^−3^ s^−1^), respectively. The *k*_3_ is approximately 23-fold larger than that of SARS-CoV M^pro^, which indicates that N3 inactivates PDCoV M^pro^ faster than it does SARS-CoV.

### 3.4. Binding of N3 to PDCoV M^pro^

**Figure 3:**
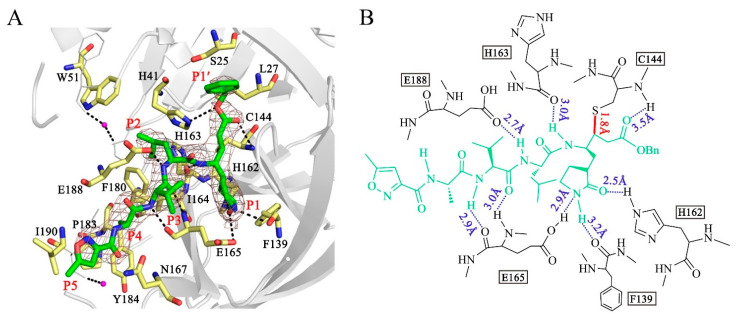
Structure of the interaction between N3 and PDCoV M^pro^. (**A**) Stereoview of N3 bound to the substrate-binding pocket of PDCoV M^pro^. N3 is colored green and shown with a simulated annealing 2mFo-DFc omit map contoured at 1.0 σ. The residues at the substrate-binding pocket that contribute to the interaction between N3 and PDCoV M^pro^ are shown in yellow. Waters are shown in magenta. The P1′, P1, P2, P3, P4 and P5 sites are labeled. (**B**) Detailed interactions between PDCoV M^pro^ and N3. N3 is shown in green. Hydrogen bonds are shown as blue dashed lines. The covalent bond formed by N3 and the Sγ atom of Cys-144 is shown as a solid red line.

In the crystal structures, N3 is bound to each protomer of the M^pro^ dimer. We thus only discuss the binding mode in one of the protomers. The inhibitor is located in the substrate-binding pocket, which adopts the canonical conformation seen in other M^pro^-N3 complex structures. As an irreversible inhibitor, the Cβ atom of the vinyl group on N3 is bound to the Sγ atom of Cys-144 through a 1.8 Å covalent bond (Figure 3A,B). The lactam ring of the glutamine analog N3 at the P1 site inserts into the S1 pocket and forms 3.2 Å, 2.5 Å and 2.9 Å hydrogen bonds with the carbonyl oxygen of Phe-139, the imidazole ring NH of His-162 and the Oε1 atom of Glu-165, respectively. The side chain of Leu at the P2 position extends into the S2 pocket and participates in hydrophobic interactions with the hydrophobic amino acids Trp, Ile and Phe. The valine side chain of N3 at the P3 position is exposed to solvent. The alanine residue in the P4 position inserts into a pocket composed of the residues Pro-183 and Tyr-184, leading to a hydrophobic interaction among these residues. The isoxazole at the P5 position, Gln-167 and Ile-190 form a “sandwich structure” by van der Waals interaction. The P2 and P4 sites insert into the S2 subsite and S4 subsite well. Moreover, the backbone NH of Cys-144, the carbonyl oxygen atoms of His-163 and Glu-165, the Oε1 atom of Glu-188 and the NH group of Glu-165 form hydrogen bonds with the inhibitor N3, which ensures tight binding between the M^pro^ and the inhibitor, as shown in Figure 3. We concluded that peptidomimetic inhibitors carrying the Michael acceptor warhead N3 are effective against the M^pro^ of PDCoV.

### 3.5. The P1′ Position May Play an Important Role in the Interaction between PDCoV M^pro^ and Inhibitors

Previously, we have designed 16 N3 derivatives that target PEDV M^pro^ (the detailed structures and their chemical synthesis were described in our previous paper) [27]. Next, we evaluated the inhibitory activity of these compounds against PDCoV M^pro^. Among these compounds, M14 and M25 exhibited stronger inhibition than N3 (Table 4). The *k*_3_/*K*_i_ values of M14 and M25 were 13.8 and 9.9, respectively, indicating that they have much better inhibitory activity than N3, which had a *k*_3_/*K*_i_ of 6.1. The detailed inhibition parameters of N3, M14 and M25 are listed in Table 5. Interestingly, we found that the three compounds shared the same side groups at all positions except the P1′ position (Table 5). The benzyl group at the P1′ position of N3 interacts with Ser-25 and Leu-27 through van der Waals forces. Therefore, we suggest that rational design of the P1′ position could dramatically enhance the interaction between the substrate-binding pocket and the inhibitor. Future modification of peptidomimetic inhibitors at the P1′ position has the potential to control acute gastroenteritis in pigs infected with PDCoV.

## 4. Discussion

The M^pro^ is an ideal target for drug design against CoVs. Since IBV, the first CoV to be described was discovered in 1937, four genera of CoVs have been identified. Currently, we have a thorough understanding of the M^pro^ structures of alpha-, beta- and gamma-CoVs; however, we know little about the *Deltacoronavirus* M^pro^. In this paper, we present the first structure of the M^pro^ of a newly emerged *Deltacoronavirus* (PDCoV) in complex with a Michael acceptor inhibitor.

**Figure 4:**
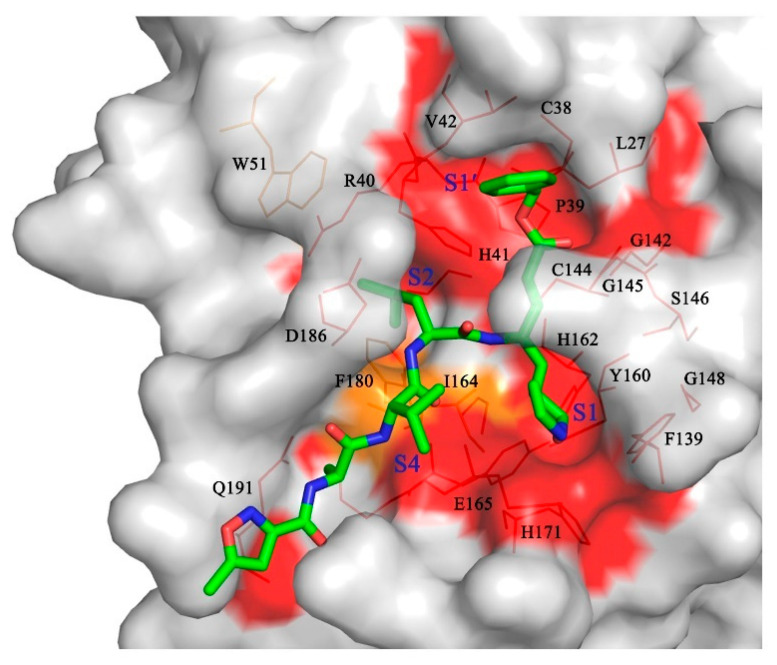
Structure-based substrate-binding pocket conservation analysis of M^pro^s from four different genera. Surface representation showing the conserved substrate-binding pockets from the ten CoV M^pro^s listed in Figure 2B. The background is PDCoV M^pro^. Red: identical residues among all ten CoV M^pro^s; orange: substituted in two CoV M^pro^s. The S1, S2, S4, and S1′ pockets and the residues that form the substrate-binding pocket are labeled. N3 is shown in green.

As observed in the previously reported M^pro^ structures, PDCoV M^pro^ presented a functional homodimer and conserved His-Cys dyads. Furthermore, a detailed comparison of the M^pro^ structures showed that PDCoV M^pro^ shares a similar overall structure and a relatively conserved substrate-binding pocket with the M^pro^s of the other three CoV genera, especially the key residues located at the S1, S4, and S2 subsites (Figure 4). Meanwhile, the irreversible inhibitor N3 in our structure, designed based on the structure of SARS M^pro^ in complex with its substrate, could inactivate PDCoV and multiple CoV M^pro^s. These results also proved the conservation of the overall structures and substrate binding pockets of M^pro^s. As demonstrated, emerging zoonotic viruses such as SARS-CoV-2, SARS-CoV and MERS-CoV are a potential threat to public health because of the existing viral variants. As an important pathogen of piglets, the nonhuman animal virus PDCoV poses the risk of cross-species transmission to humans as well [30,31]. Therefore, the conserved CoV M^pro^ we identified could be considered a drug target in the event of genetic changes during human-to-human or animal-to-human transmission of CoVs.

**Figure 5:**
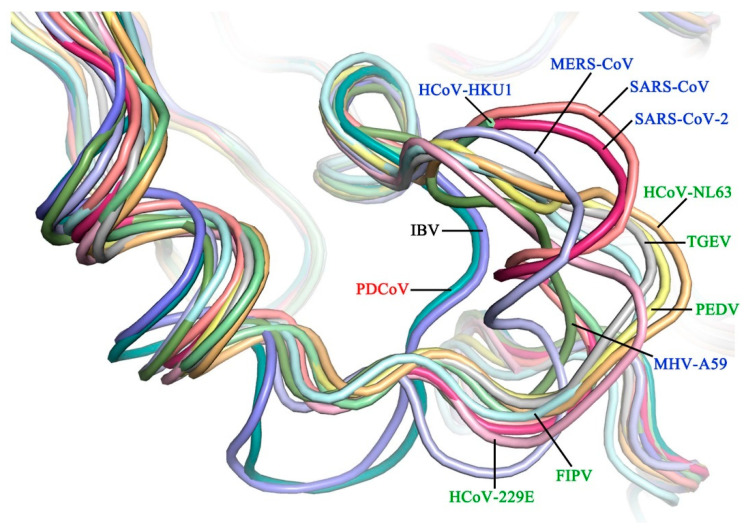
Structures of the backbone of a loop containing residues 41–51 in M^pro^s from four different genera (PDCoV, deep teal; HCoV-NL63, light orange; HCoV-229E, light pink; FIPV, pale cyan; PEDV, pale yellow; TGEV, silver; SARS-CoV-2, warm pink; SARS-CoV, salmon; MERS-CoV, light blue; HCoV-HKU1, pale green; MHV-A59, sumudge; IBV, slate).

Interestingly, the structure and conformation of M^pro^s presented a stable characteristic evolution and obvious species correlation. We superposed the determined structures of M^pro^s from CoVs in four different genera and found some loops, especially for the region from 41–51, that exhibited corresponding features (Figure 5). For example, this loop in *Alphacoronavirus* and *Betacoronavirus* forms a 3_10_ helix, while in *Gammacoronavirus* IBV, it forms a short loop. Surprisingly, the loop from residues 41–51 of PDCoV M^pro^ adopts a conformation similar to that of IBV M^pro^, which supports that the *Deltacoronavirus* may be closely related to *Gammacoronavirus* [6]. Since the loop from 41–51 is associated with the outer wall of the S2 pocket, our structural information will support reasonable broad-spectrum peptidomimetic drug design based on the evolutionary conservation of M^pro^s from CoVs.

The peptidomimetic inhibitor N3, which carries a Michael acceptor warhead, was also effective against M^pro^ of PDCoV, the newly emerging *Deltacoronavirus*. Peptidomimetic compounds are attractive inhibitors for the development of novel antiviral therapies. These compounds target proteases that are essential for viral replication. For example, boceprevir, telaprevir and simeprevir are peptidomimetic drugs that act as viral NS3/4A serine protease inhibitors of hepatitis C virus (HCV) [43,44,45], while saquinavir, indinavir, nelfinavir, ritonavir, and amprenavir are clinically approved human immunodeficiency virus (HIV) protease inhibitors, which have a similar molecular structure to the protease substrate [46,47]. Furthermore, these peptidomimetic drugs were derived from lead compounds identified based on viral protease structures. For instance, boceprevir, which is a tripeptide derivative that forms a covalent bond with Ser-139 to inactivate the NS3/4A protease [45], was designed based on an undecapeptide alpha-ketoamide inhibitor identified from compound libraries. Hence, after multiple rounds of modification, the inhibitor N3 is a currently available compound for broad-spectrum drug design. It is worth noting that P1′ may be a key position of compound modification for broad-spectrum drug design because of the side chains variability in the amino acid at position 25, which directly participates in the interaction with the inhibitor. In our study, both N3 derivatives (M14 and M25) with improved inhibitory activity against PDCoV M^pro^ presented a unique group at P1′. Therefore, it is necessary to balance the relatively conserved substrate binding pockets during rational drug design. Furthermore, we found that M25 exhibited potent inhibition of both PDCoV and PEDV M^pro^ proteins [27]. The two main emerging swine CoVs, PDCoV and PEDV, account for the majority of lethal watery diarrhea in neonatal pigs in the past decade. More recently, the epidemiological evidence shows that the rate of PDCoV coinfection with PEDV has increased up to 51% in China [32,48]. Therefore, M25 could be further developed to combat both PDCoV and PEDV infection in the swine industry.

In summary, the structure of PDCoV M^pro^ in complex with the Michael acceptor inhibitor N3 provides a basis for the inactivation of *Deltacoronavirus* viral proteases. The structural comparison of different viral enzymes identified a conserved substrate-binding pocket in all CoV M^pro^s; this pocket is a target for the development of broad-spectrum antivirals against all existing and emerging CoVs.

## Figures and Tables

**Table 1:** X-ray data-processing and refinement statistics.

Statistics	Value for the Porcine deltacoronavirus (PDCoV) main protease (M^pro^)-N3 Complex
**Data collection**	
Wavelength (Å)	0.97923
Resolution limit (Å)	50.00–2.60 (2.64–2.60)
Space group	*P*6_1_
Cell parameters	
*a* (Å)	122.29
*b* (Å)	122.29
*c* (Å)	289.75
α, β, γ (°)	90, 90, 120
Total no. of reflections	75,173 (3733)
No. of unique reflections	74,737 (7392)
Completeness (%)	99.6 (99.3)
Redundancy	20.5 (20.3)
*R*_merge_ (%)	17.2 (>100)
Sigma cutoff	0
*I/σ(I)*	12.4 (1.1)
**Refinement**	
Resolution range (Å)	49.74–2.60
*R*_work_ (%)	19.21
*R*_free_^a^ (%)	24.14
No. atoms	
Protein	14,122
Water	461
Ligands	294
*B*-factors (Å^2^)	
Protein	57.29
Water	47.07
Ligands	69.01
R.m.s. deviations	
Bond lengths (Å)	0.005
Bond angles (°)	0.72
Ramachandran	
Favored (%)	96.05
Allowed (%)	3.95
Outliers (%)	0.00

^a^*R*_free_ was calculated with 5% of the reflection data. Refinement statistics were calculated with the table one utility of PHENIX.

**Table 2:** The root-mean-square deviation (RMSD) values of domain I and domain II or domain III between the structures of PDCoV M^pro^ and M^pro^s from three other CoV genera.

Genus	Coronaviruses	RMSD (Å) (Domain I and Domain II)	RMSD (Å) (Domain III)	PDB ID	References
*Alphacoronavirus*	HCoV-NL63	1.6	2.1	5GWY	[26]
	HCoV-229E	1.5	1.7	2ZU2	[24]
	FIPV	1.5	1.6	5EU8	[25]
	PEDV	1.4	1.7	5GWZ	[27]
	TGEV	1.7	1.6	2AMP	[15]
*Betacoronavirus*	SARS-CoV-2	1.3	1.9	6LU7	[23]
	SARS-CoV	1.3	2.1	2AMQ	[15]
	MERS-CoV	1.3	1.6	5C3N	[22]
	HCoV-HKU1	1.4	1.6	3D23	[29]
	MHV-A59	1.3	1.5	6JIJ	[21]
*Gammacoronavirus*	IBV	1.1	1.4	2Q6F	[28]

Human coronavirus NL63, HCoV-NL63; Human coronavirus 229E, HCoV-229E; Feline infectious peritonitis virus, FIPV; Porcine epidemic diarrhea virus, PEDV; Transmissible gastroenteritis virus, TGEV; Severe acute respiratory syndrome coronavirus 2, SARS-CoV-2; Severe acute respiratory syndrome coronavirus, SARS-CoV; Middle East respiratory syndrome coronavirus, MERS-CoV; Human coronavirus HKU1, HCoV-HKU1; Mouse hepatitis virus A59, MHV-A59; Infectious bronchitis virus, IBV.

**Table 3:** The *K*_m_ and *k*_cat_ values of the representative M^pro^s from different CoVs.

Genus	Coronaviruses	*K*_m_ (µM)	*k*_cat_ (s^−1^)	References
*Deltacoronavirus*	PDCoV	56.6 ± 1.9	0.030 ± 0.009	In this study
*Alphacoronavirus*	HCoV-NL63	50.8± 3.4	0.098 ± 0.004	[26]
	HCoV-229E	29.8 ± 0.9	1.27 ± 0.09	[15]
	FIPV	13.5 ± 1.8	0.6 ± 0.06	[15]
	TGEV	61 ± 5	1.39 ± 0.09	[15]
*Betacoronavirus*	SARS-CoV	129 ± 7	0.14 ± 0.01	[15]
	HCoV-HKU1	83.2 ± 13.3	1.1 ± 0.12	[29]
	MHV-A59	77 ± 5	0.083 ± 0.006	[15]
*Gammacoronavirus*	IBV	139 ± 15	0.22 ± 0.03	[15]

Porcine deltacoronavirus, PDCoV; Human coronavirus NL63, HCoV-NL63; Human coronavirus 229E, HCoV-229E; Feline infectious peritonitis virus, FIPV; Transmissible gastroenteritis virus, TGEV; Severe acute respiratory syndrome coronavirus, SARS-CoV; Human coronavirus HKU1, HCoV-HKU1; Mouse hepatitis virus A59, MHV-A59; Infectious bronchitis virus, IBV.

**Table 4:** Evaluation of the inhibitory activity of compounds targeting PDCoV M^pro^. The inhibition ratio (Ir) is defined as the percent inactivation of the initial enzymatic activity of PDCoV M^pro^.

Compound	Inhibition Ratio (Ir)	Inhibitory Activity ^a^
N3	62%	+ + +
M1 ^c^	37%	+ +
M2 ^c^	53%	+ + +
M3 ^c^	21%	+
M5 ^c^	-	N/A ^b^
M6 ^c^	-	N/A
M7 ^c^	30%	+ +
M8 ^c^	20%	+
M10 ^c^	62%	+ + +
M11 ^c^	23%	+
M12 ^c^	27%	+
M13 ^c^	21%	+
M14 ^c^	82%	+ + + + +
M17 ^c^	39%	+ +
M18 ^c^	50%	+ + +
M19 ^c^	53%	+ + +
M25 ^c^	74%	+ + + +

^a^ Percentage inhibitory activity: + + + + +, >80%; + + + +, 70%−80%; + + +, 50%−70%; + +, 30%−50%; +, <30%. ^b^ No inhibition was observed. ^c^ The detailed structures and chemical synthesis of the compounds were described in reference [27].

**Table 5:** Enzyme inhibition data for inhibitors of PDCoV M^pro^.

Compound	Structure	*K*_i_ (µM)	*k*_3_ (10^−3^s^−1^)	*k*_3_/*K*_i_
N3	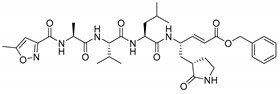	11.98 ± 0.13	72.9 ± 7.1	6.1
M14	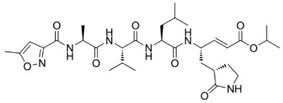	7.56 ± 0.26	104.4 ± 2.3	13.8
M25	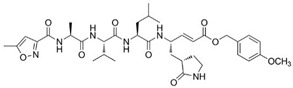	8.80 ± 0.15	86.8 ± 5.1	9.9

## Data Availability

Atomic coordinates for the crystal structure of PDCoV M^pro^ in complex with N3 can be accessed using PDB code 7WKU in the RCSB Protein Data Bank (https://doi.org/10.2210/pdb7WKU/pdb accessed on 25 January 2022). Authors will release the atomic coordinates and experimental data upon article publication.
